# Case report: Unilateral optic nerve aplasia and developmental hemi-chiasmal dysplasia with VEP misrouting

**DOI:** 10.1007/s10633-020-09788-7

**Published:** 2020-08-27

**Authors:** Sian E. Handley, Oliver R. Marmoy, Sri K. Gore, Kshitij Mankad, Dorothy A. Thompson

**Affiliations:** 1 Clinical and Academic Department of Ophthalmology, Great Ormond Street Hospital for Children, Great Ormond Street, London, WC1N 3JH UK; 2 UCL Great Ormond Street Institute of Child Health, University College London, 30 Guildford Street, London, WC1N 1EH UK; 3 Manchester Metropolitan University, Manchester, UK

**Keywords:** Chiasm, Hemi-chiasm, Misrouting, Optic nerve aplasia, Visual evoked potential

## Abstract

**Purpose:**

To describe the trans-occipital asymmetries of pattern and flash visual evoked potentials (VEPs), in an infant with MRI findings of unilateral optic nerve aplasia and hemi-chiasm dysplasia.

**Methods:**

A child with suspected left cystic microphthalmia, left microcornea, left unilateral optic nerve aplasia, and hemi-chiasm underwent a multi-channel VEP assessment with pattern reversal, pattern onset, and flash stimulation at the age of 16 weeks.

**Results:**

There was no VEP evidence of any post-retinal visual pathway activation from left eye with optic nerve aplasia. The VEP trans-occipital distribution from the functional right eye was skewed markedly across the midline, in keeping with significant misrouting of optic nerve fibres at the chiasm. This was supported by the anatomical trajectory of the optic chiasm and tracts seen on MRI.

**Conclusion:**

This infant has chiasmal misrouting in association with unilateral optic nerve aplasia and unilateral microphthalmos. Chiasmal misrouting has not been found in patients with microphthalmos or anophthalmos, but has been reported after early eye loss in animal models. Our findings contribute to our understanding of the discrepancy between the visual pathway physiology of human unilateral microphthalmia and animal models.

## Introduction

In humans, fully functional binocular vision relies upon the correct formation of the optic chiasm. At the chiasm, a designated proportion of retinal ganglion cell axons from the nasal retina of each eye must project across the midline to innervate the contralateral hemisphere, representing the bi-temporal field. The remaining proportion of fibres from the temporal retina of each eye project to the ipsilateral hemisphere and represent the bi-nasal fields. The correct development of these crossing and non-crossing chiasmal pathways relies upon the temporo-spatial and dosage-dependent interaction of many guidance cues [1, 2]. The two major conditions associated with a disproportion of fibres at the chiasm are chiasmal aplasia or hypoplasia (non-decussating retinal fugal fibre syndrome) [3] in which there is absent or markedly reduced chiasmal crossing, and albinism in which there is proportionally increased chiasmal crossing, referred to as chiasmal misrouting. Chiasmal misrouting has also been described in conditions distinct from albinism, such as FHONDA syndrome [4].

The lateralization of flash, pattern reversal, and pattern onset visual evoked potential (VEP) distributions across the occiput is used to investigate chiasmal and hemisphere function in humans. VEPs are typically largest at the occipital midline (Oz) with smaller waveforms of similar size recorded over the left and right occipital scalp (Fig. 4—schematic). This horizontal symmetric VEP distribution about the midline depends on stimulation of both occipital hemispheres; asymmetric VEP distributions are typically recorded to hemi-field stimulation in normal subjects. The main positive peak of monocular flash VEPs recorded with a mid-frontal (Fz) reference has the same lateralized trans-occipital distribution as monocular pattern reversal VEPs, demonstrated in patients with well-defined unilateral occipital lesions [5, 6]. These studies suggest that a trans-occipital difference in peak time of 6 ms or an amplitude reduction of 50% of a flash VEP indicates a hemianopic field defect [7, 8]. Full field pattern reversal VEPs recorded with a mid-frontal reference to large check widths (i.e. 50′) lateralize paradoxically: the positive peak is largest over the hemisphere opposite to the functional hemisphere [9, 10]. Pattern onset VEPs positive peaks, in contrast, are maximal over the functional ipsilateral hemisphere [11, 12]. These contrasting VEP distributions have been used to investigate developmental chiasmal malformations in humans including achiasmia and albinism [13–15].

Unilateral disruption to the development of one eye, such as microphthalmia or anophthalmia, occurs within the first few weeks after conception. The way chiasmal development is disrupted when one eye fails to develop is dependent upon the species of mammal. For example, the enucleation of one eye of a rodent or ferret early in development appears to increase the number of crossing fibres at the chiasm from the remaining eye [16], whereas human patients with anophthalmia and severe microphthalmia have not shown any VEP evidence of a change in the distribution of crossing and non-crossing fibres [16]. Achiasmia, however, has been reported in an infant with unilateral optic nerve hypoplasia [17].

Microphthalmia, anophthalmia, and coloboma (MAC) are related structural congenital ocular malformations [18]. Orbital cysts can occur as part of the MAC spectrum as separate structures or as part of the eye itself [19]. Microphthalmia has been described with optic nerve and chiasmal hypoplasia [18, 20]. The MAC spectrum can be as extreme as microphthalmia with associated complete absence of the optic nerve (aplasia) [21]. We describe an infant with unilateral microphthalmia and optic nerve aplasia who had VEP and radiological evidence of chiasmal misrouting, with excessive crossing of typically ‘non-crossing’ chiasmal fibres.

## Patient

A 3-month-old girl was referred to a tertiary eye hospital for management of her left microphthalmos. She was born at 37 weeks by caesarean section weighing 1.26 kg following a complicated pregnancy. Her mother had experienced moderate reflux and recurrent gastritis as well as one episode of gastroenteritis during the tenth week of pregnancy; she did not suffer infection or nutritional deficiency. There was no history of medication use during pregnancy. The mother was monitored intensively during the third trimester for intrauterine growth retardation. The patient spent 3 weeks of the post-natal period in a special care baby unit for weight gain, requiring minimal respiratory support and an incubator for 3 days.

At term corrected age, her left eye appeared microphthalmic with an unusual fundal appearance, whilst her right eye appeared normal. Three months later, she was not showing any reproducible fix and follow responses with either eye, she had a reduced gaze preference to the left, but no abnormal eye movements were noted. There was an afferent pupil defect of the microphthalmic left eye with minimal pupil constriction to light. Both corneas and lenses were clear; irides were brown and well pigmented. Her skin and fundus were also appropriately pigmented. Her left eye appeared clinically microphthalmic with a microcornea as the central horizontal corneal diameter measured 7.5 mm (reference range of 9.0–10.5 mm [22]).

Dilated fundus examination of the left eye revealed a large nasal chorio-retinal coloboma, a rudimentary ‘optic nerve head' and no discernible macular reflex or foveal pit. The right eye appeared structurally normal with a healthy disc, normal macular appearance, and normal horizontal corneal diameter.

**Fig. 1:**
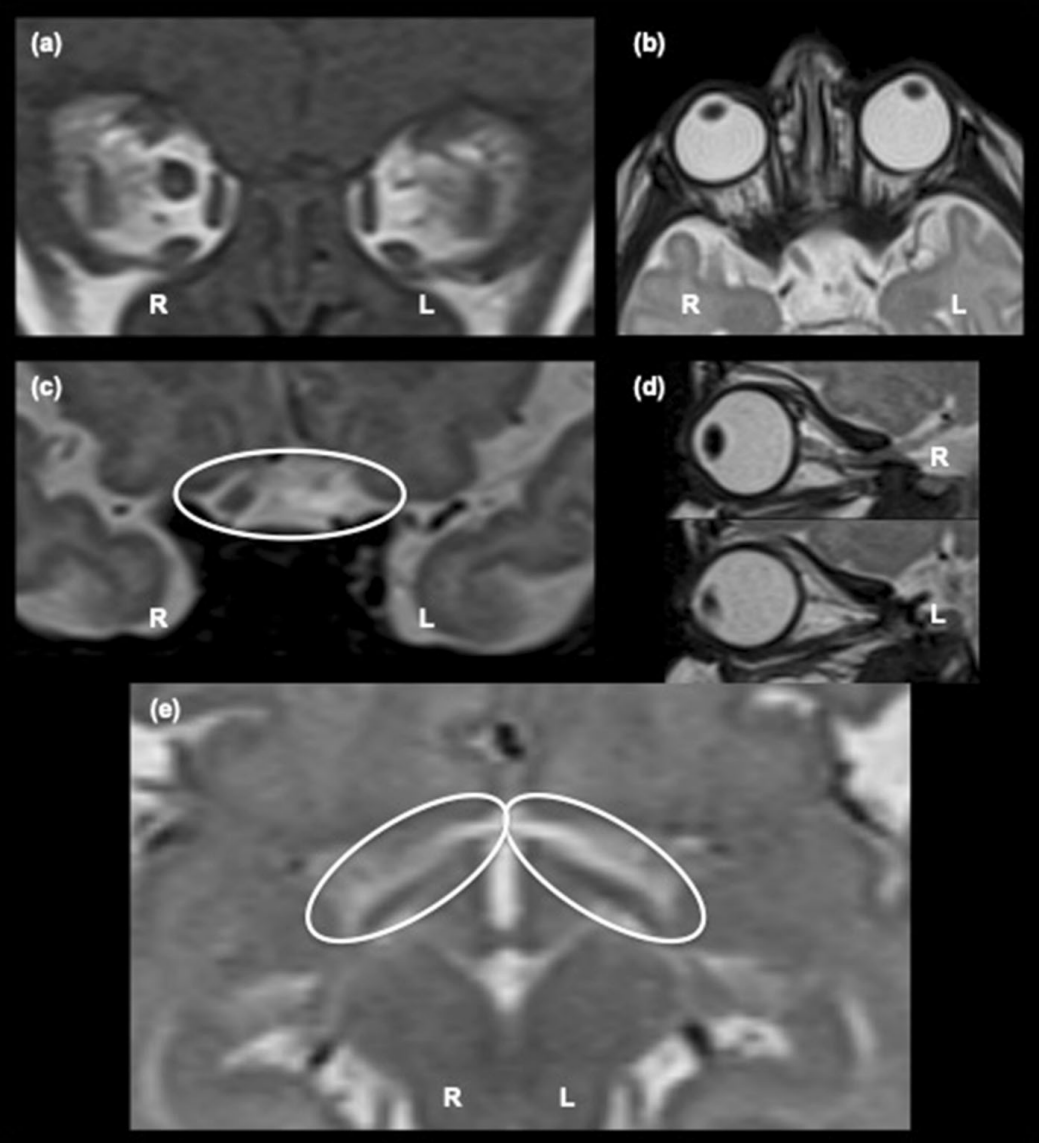
A composite of MRI images from the patient with right (R) and left (L) sides of the patient marked. The left optic nerve head cannot be detected at the back of the eye (**a**) and is grossly hypoplastic within the orbit (**b**, **d**). As the nerves track backwards towards the chiasm, only the right optic nerve is visible pre-chiasmally (**c** circled). Post-chiasm, there is a marked asymmetry in the distribution of the optic tracts (**e**), which are thicker on the left side compared to the right (circled)

Unexpectedly, the ultrasound b-scan measured an axial length of 15.3 mm right eye and 15.7 mm left eye. This was supported by the MRI of the brain and orbits (Fig. 1), which showed a longer globe length of the left compared to the right eye (Fig. 1b). It was concluded that the left eye was within the spectrum of microphthalmia with a cystic expansion of the posterior segment resulting in these similar axial lengths.

This MRI was undertaken at 10 weeks of age. The left optic nerve head was not evident on MRI (Fig. 1a). The left eye appeared to have a rudimentary nerve (Fig. 1d), but this rudimentary optic nerve did not approach the chiasm (Fig. 1c). The right eye optic nerve appeared normal within the orbit and was visible at the chiasm. The chiasm was described as hemi-hypoplastic on the left side. The post-chiasmic visual pathway showed a marked asymmetry of the optic tracts, and the left hemisphere optic tracts were considerably thicker than in the right hemisphere (Fig. 1e). No other focal, or diffuse, abnormality of the cerebral hemispheres or brainstem was detected. The cerebral spinal fluid spaces were unremarkable with no restricted diffusion. At the age of 16 weeks, 4 months, the patient had a behavioural vision assessment and visual electrophysiology tests.

## Methods

Behavioural vision was assessed using fix and follow to a range of graded toy sizes. Visual fields to confrontation were assessed using a two-examiner technique by an experienced paediatric orthoptist, unaware of the clinical history or underlying diagnosis.

The patient underwent VEP testing as per the department protocol adhering to the ISCEV VEP standard [23]. Checkerboards of five sizes (check widths range 25′–400′), which included the ISCEV standard large checks (50′), were presented. Electrodes were positioned in an array across the occipital scalp at O1 (left) Oz (mid) and O2 (right) all referred to Fz (mid-frontal). Skin electrodes were placed along the infra-orbital rims for flash electroretinogram (ERG) recording. Impedance was equal and maintained at below 5 kΩ. Pattern reversal checkerboard stimuli were presented at three reversals per second. Pattern onset stimuli were presented for 230 ms followed by a uniform grey field of equal luminance for 330 ms. Checkerboard stimuli were presented in on a plasma display screen with 97% Michelson contrast. The plasma display of mean luminance of 82 cd/m^2^ was centred at the patient’s eye level subtending 30° at a 1 m viewing distance. Fixation accuracy was monitored via a close circuit TV system, and data acquisition was paused if any fixation loss was seen. A handheld strobe (Grass model PS33) was held at 30 cm to present flash stimuli at an intensity setting 4 (time integrated luminance of 8 cd s/m^2^) at a stimulation rate of 3 Hz. Simultaneous flash ERGs and VEPs were produced by flashes presented when both eyes were open and to each eye in turn by occlusion. Throughout recordings, a minimum of two trials were recorded to ensure reproducibility to each stimulus before a grand average was created.

The VEP waveforms from the right and left occiput were overlaid from each stimulus to examine the trans-occipital asymmetries.

## Results

The patient’s visual acuity at 16 weeks of age had improved from initial and 10 week assessments. She now demonstrated fixing and following to 2″ toys with both eyes open and monocularly with her right eye. Fixation from the right eye was steady and central, but during monocular left eye testing no consistent fixation was seen. No nystagmus was seen when both eyes were open. However, her left half field to confrontation visual fields testing was reproducibly reduced .

**Fig. 2:**
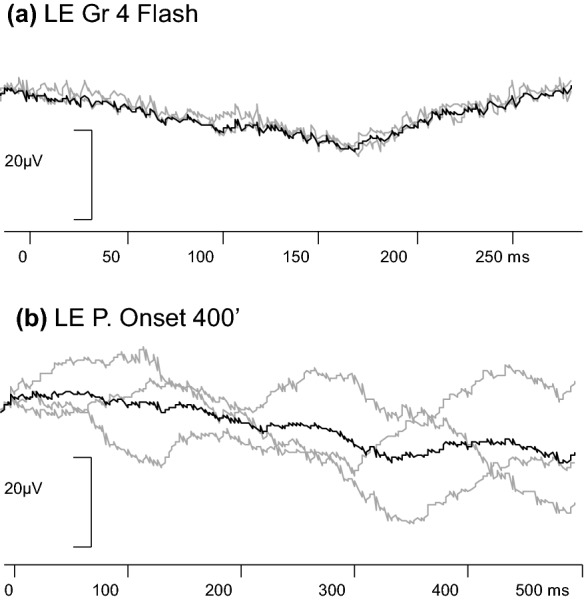
Left eye (LE) visual evoked potential (VEP) traces produced by flash (**a**) and large check pattern (P) onset (**b**) stimulation. No responses were evident consistently above the levels of background noise. Individual trials are shown in grey and the grand average in black

**Fig. 3:**
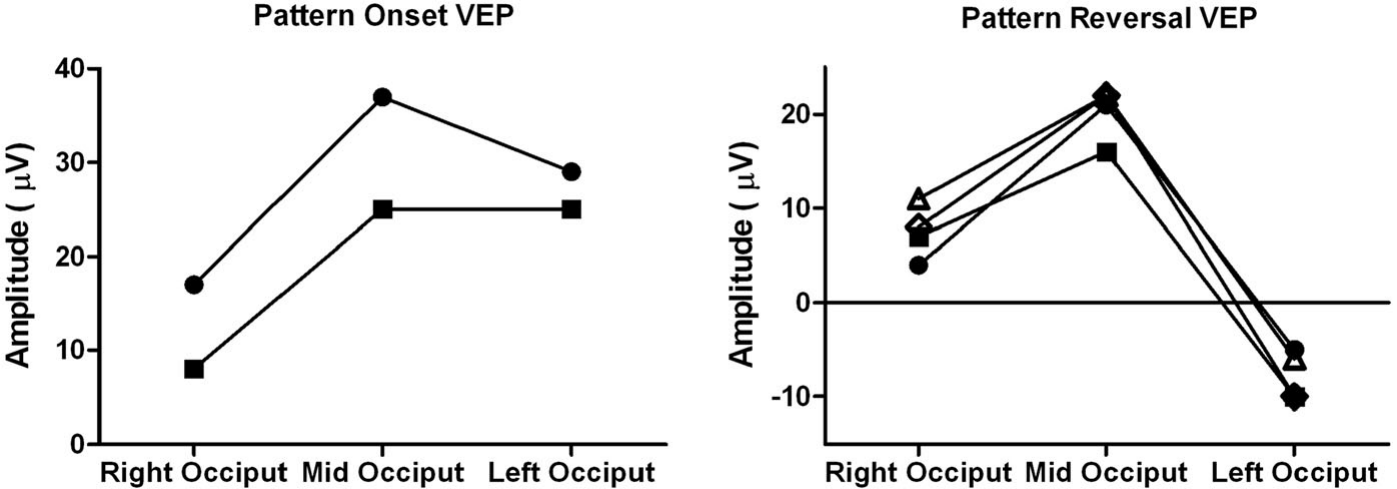
Amplitude of all pattern visual evoked potentials (VEPs) across the occiput is shown. Filled square = 25′, filled circle = 50′, open diamond = 100′, and open triangle = 200′. This demonstrates the consistency in the distribution of the pattern VEPs with changes in check size; onset VEPs are smaller on the right occiput whilst reversal VEPs are smaller, actually negative, on the left occiput

Skin ERGs of similar b-wave time to peak were recorded from each eye during flash VEP recordings. The ERG was smaller in amplitude from the colobomatous and microphthalmic left eye. Despite very good co-operation during monocular VEP stimulation, no consistent flash or pattern onset VEP (to large check widths 400′ and 200′) was evident from the open left eye (Fig. 2). The measurements of the VEPs produced with both eyes open to patterned and flash stimuli are displayed in Fig. 3. With both eyes open, pattern reversal VEPs were evident to 200′, 100′, 50′, and 25′ check widths. The amplitudes and peak times of the pattern reversal VEPs over the mid-occiput (Fig. 3) were appropriate for her corrected age [24].

**Fig. 4:**
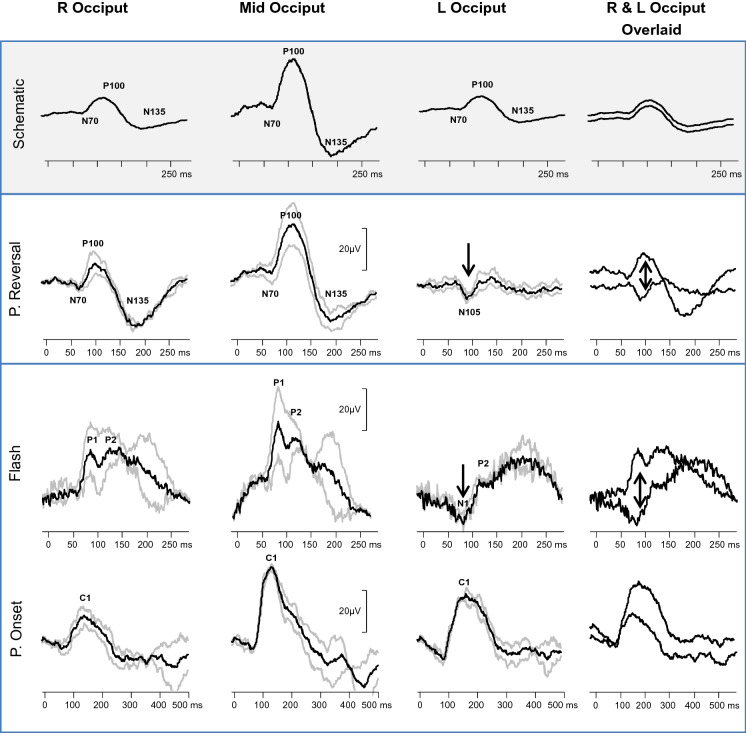
Pattern (P) reversal, pattern onset, and flash visual evoked waveforms (VEPs) from right (R), mid- and left (L) occiput. Individual trials are shown in grey, whilst the grand average is in black. The waveforms from the right and left occiput are also displayed superimposed to highlight the trans-occipital asymmetry. Note how the distribution of the pattern reversal and the flash VEP waveforms is the same with a negativity at around 80 ms over the left occiput (black arrow). In contrast, VEP to pattern onset stimulation is relatively smaller over the right occiput. A schematic illustration of typical symmetric pattern reversal VEP distribution is shown for comparison

Flash and pattern reversal VEPs showed similar lateralization of the major positive peak larger over the right occiput. Both showed contralateral negativity. The reversal VEPs were negative over the left occiput at a corresponding time to the positive peak over the right occiput (Figs. 3, 4). This occipital distribution is consistent with the description of paradoxical lateralization of right half field pattern reversal VEPs from the left occipital hemisphere [9, 10]. The pattern onset VEPs showed the opposite distribution with a larger positivity detected over the left occiput compared to the right occiput (Figs. 3, 4).

## Discussion

The lack of post-retinal pathway activation from the clinically microphthalmic and colobomatous left eye was unsurprising, but the proportional overcrossing of fibres at the chiasm from the unaffected right eye was unexpected. We found both structural and functional evidence of chiasmal misrouting from MRI and the occipital distributions of the VEP.

The lateralized VEPs in this patient were typical of those produced when only the left occipital hemisphere is stimulated. Specifically, the pattern reversal and flash VEPs lateralize paradoxically, whilst pattern onset VEPs do not (Figs. 3, 4), as previously reported [9, 11]. The nature and extent of the trans-occipital asymmetry were corroborated by the distribution of pattern VEPs to each of different check widths used (Fig. 3). Such building of supporting VEP evidence by more than one check width is advantageous, particularly in children (see also [25]).

Pattern onset and pattern reversal VEPs in this infant showed trans-occipital amplitude differences of more than 50%, consistent with homonymous hemianopia [8]. The trans-occipital asymmetry of flash VEP amplitudes was not as large, but clearly exceeded the cited clinical decision minimum of 20% [26]. The overall pattern VEP distributions are similar to those reported in human albinism. In infants with chiasmal misrouting, flash VEPs typically show more marked trans-occipital asymmetry than onset VEPs; towards the age of 7 years, onset VEPs typically show a clearer asymmetry than flash VEPs [13, 27]. Thus, our patient reinforces the value of full investigation with flash and a range of pattern stimuli to fully investigate visual pathway misrouting.

With both eyes open, the extent of the left hemi-field to confrontation tests was consistently reduced, suggesting a left hemianopic visual field deficit of the only seeing (right) eye. Given the patient’s young age together with loss of contribution of her left eye to her functional binocular field, field restriction cannot be considered conclusive. A marked loss of the left visual field is unexpected, given that monocular visual fields are full in patients with human albinism [17, 28]. However, bi-temporal hemianopia has been reported in a few, but not all, cases of human achiasmia [14, 29].

The pathogenesis of the unilateral misrouting from the only functional eye might involve an imbalance or interaction of events such as mutation of genes regulating chiasmal development and left–right asymmetry [4], or a lack of competition at the chiasm from the left eye ganglion cell axons facilitating an overcrossing of right eye fibres. Although overcrossing is reported in murine models, this has not yet been reported in larger studies of human unilateral microphthalmic or anophthalmic subjects [16].

The hemi-hypoplastic chiasm in our case was rotationally skewed along the transverse axial plane (the right side of the anterior chiasm more anterior than the left). Interestingly, an unusual vertical chiasmal orientation has been reported also in bilateral microphthalmia with cyst [20]. This suggests possible mechanical interaction between the microphthalmia/cyst, coloboma, and the orientation of nerve growth towards the chiasm.

The incidence and genetics of unilateral or bilateral optic nerve aplasia with microphthalmos are unknown. Sporadic, bilateral cases of optic nerve aplasia have been associated with an overexpression of *PAX6*, a developmental control gene, which affects retinal ganglion cell guidance [30, 31]. Dominant expression of bilateral optic nerve aplasia has been proposed due to haploinsufficiency of *CYP26A1* and *CYP26C1* genes that encode retinoic acid (RA) degrading enzymes [32]. RA is important for molecular regulation of neural crest cells that form many ocular tissues [30, 32].

Our findings suggest the absence of an optic nerve alters the balance of chiasmal guidance behaviour, though a lack of crossing inhibition or excessive crossing guidance, and results in too much crossing. Midline crossing by retinal ganglion cell axons is facilitated by overexpression of Neuropilin-1, VEGF-A, NrCAM, and PlexinA1 and prevented by under underexpression EphB1, Zic2, Foxd1, and EphrinB2 [33–35]. The Sonic Hedgehog (SHH) signalling pathway orchestrates chiasmal development [36]. Mutations affecting the SHH pathway have been associated with milder ocular developmental anomalies including microcornea, microphthalmia, coloboma, and malposition of the optic nerve [36]. As the mother’s history was negative for any medications during pregnancy, a teratogen drug effect during gestation is unlikely.

The MRI and VEP evidence of chiasmal misrouting in our patient with unilateral optic nerve aplasia is in keeping with one other report [37], as is the otherwise normal brain MRI. CNS abnormalities more often occur in bilateral cases [21]. Our case highlights the co-occurrence of optic nerve aplasia and microphthalmos, which is thought to under reported, because not all microphthalmia patients undergo neuroimaging [16, 25].

The electrophysiological information significantly benefits plans for neurodevelopmental support and visual rehabilitation in these young children and their families. This baseline information will underpin our future knowledge about natural history of visual adaptation in such patients.

## Conclusion

Chiasmal misrouting in an infant with unilateral microphthalmia and unilateral optic nerve aplasia provides further evidence [37] that optic nerve aplasia may alter the ganglion cell axon guidance trajectory through the chiasm. This unexpected finding contributes to the phenotype and supports neuroimaging and VEP assessment of microphthalmic patients [21, 37].
